# Genetics and Epigenetics in Complex Diseases

**DOI:** 10.3390/ijms24098186

**Published:** 2023-05-03

**Authors:** Elixabet Lopez-Lopez

**Affiliations:** 1 Department of Biochemistry and Molecular Biology, University of the Basque Country, UPV/EHU, 48940 Leioa, Spain; elixabet.lopez@ehu.eus; 2 Pediatric Oncology Group, BioCruces Bizkaia Health Research Institute, 48903 Barakaldo, Spain

Many of the most common diseases are influenced by a combination of multiple factors, which include environmental effectors, as well as genetic and epigenetic variations. Therefore, these diseases are grouped under the term “complex” diseases because, from the point of view of genetics, they cannot be explained by simple Mendelian inheritance.

The aim of this Special Issue was to identify genetic and epigenetic factors involved in such diseases, in order to improve not only the knowledge of risk factors for those diseases, which could be of help for prevention, but also to improve the understanding and characterization of each disease, and to optimize and personalize their treatment.

On the one hand, seven research articles identified genetic and epigenetic variations of relevance for pediatric and adult malignancies, coronary artery disease, body shape and metabolic traits, and Alzheimer’s Disease.

In particular, two articles focused on pediatric malignancies. While Schedel et al. identified a germline variant in *RAD21* that can predispose to childhood lymphoblastic leukemia or lymphoma without displaying a Cornelia-de-Lange syndrome phenotype [1], Michler et al. identified a germline variant in *POT1* in a child with acute myeloid leukemia and showed a connection between this variant and POT expression and telomeric dysregulation [2]. Regarding adult malignancies, Pacholewska et al. identified local decreases in methylation levels in chronic lymphocytic leukemia patients harboring mutations at *SF3B1*, mostly in proximity to telomeric regions, enriched in cancer-related signaling genes [3], and Campos Gudiño et al. observed that SKP1, CUL1, and F-box protein complex member genes were frequently altered at the genetic and epigenetic levels in many cancer types, which might contribute to the development and progression of these malignancies [4].

Regarding other disease entities, Chou et al. proposed that combining resistin and sST2 levels with weighted genetic risk scores of *RETN* and *IL1RL1* could be helpful for the prediction of outcome in coronary artery disease [5]. Moreover, Wu et al. showed that genetic and epigenetic variations of *KLF14* were associated with body shape indices, metabolic traits, insulin resistance, and metabolically healthy status, effects that were mediated by age, sex and obesity [6]. Finally, Tortora et al. hypothesized that a polymorphism in *CD33* could be a risk factor for Alzheimer’s disease, through the binding of sialic acid, acting as an enhancer of the CD33 inhibitory effects on amyloid plaque degradation, based on in silico analyses [7].

On the other hand, four articles reviewed the literature on the role of genetic variants in vitamin D-binding protein-related diseases, psoriatic disease, vasovagal syncope, and virus-induced epigenetic changes that lead to carcinogenesis.

First, Rozmus et al. reviewed the relationship between polymorphisms in the VDBP gene which might lead to vitamin D deficiencies and diseases such as diabetes, polycystic ovarian syndrome, metabolic syndrome, or Parkinson’s disease [8]. Secondly, Queiro et al. discussed the association of genetic variants in the NF-κB pathway with the risk of suffering psoriatic disease, as well as with the comorbidities that frequently accompany it, and their relevance for improving treatment selection [9]. Then, Matveeva et al. summarized data on the genetics of vasovagal syncope, describing the inheritance pattern of the disorder, candidate gene association studies and genome-wide studies [10]. Lastly, Pietropaolo et al. reviewed the role of epigenetic changes that take place in the host cells in virus-induced cancers [11].

As a whole, this Special Issue covers different aspects of genetic and epigenetic variation with a role in different complex diseases, sheds light on possible pathways that lead to their involvement in these diseases, and suggests possible applications of such knowledge.
